# Challenges of Dissolution Methods Development for Soft Gelatin Capsules

**DOI:** 10.3390/pharmaceutics13020214

**Published:** 2021-02-04

**Authors:** Festo Damian, Mohammad Harati, Jeff Schwartzenhauer, Owen Van Cauwenberghe, Shawn D. Wettig

**Affiliations:** 1 School of Pharmacy, University of Waterloo, 200 University Ave W, Waterloo, ON N2L 3G1, Canada; fdamian@uwaterloo.ca (F.D.); ovancauwenberghe@uwaterloo.ca (O.V.C.); 2 Department of Natural Sciences, University of Michigan, 2209 Natural Sciences Building, 4901 Evergreen Rd, Dearborn, MI 48128, USA; mharati@umich.edu; 3 Catalent Pharma Solutions, 2125 Ambassador Dr, Windsor, ON N9C 3R5, Canada; Jeff.Schwartzenhauer@catalent.com; 4 Bio Therapeutic Molecules Inc., 70 Southgate Drive, Unit 4, Guelph, ON N1G 4P5, Canada; 5 Waterloo Institute for Nanotechnology, University of Waterloo, 200 University Ave W, Waterloo, ON N2L 3G1, Canada

**Keywords:** soft gelatin capsules, drug stability, poorly soluble drugs, gelatin cross-linking, solubility, dissolution method development, two-tier dissolution testing, enzymes in dissolution, surfactants

## Abstract

Recently, the development of soft gelatin capsules (SGCs) dosage forms has attracted a great deal of interest in the oral delivery of poorly water-soluble drugs. This is attributed to the increased number of poorly soluble drugs in the pipeline, and hence the challenges of finding innovative ways of developing bioavailable and stable dosage forms. Encapsulation of these drugs into SGCs is one of the approaches that is utilized to deliver the active ingredients to the systemic circulation to overcome certain formulation hurdles. Once formulated, encapsulated drugs in the form of SGCs require suitable in vitro dissolution test methods to ensure drug product quality and performance. This review focuses on challenges facing dissolution test method development for SGCs. A brief discussion of the physicochemical and formulation factors that affect the dissolution properties of SGCs will be highlighted. Likewise, the influence of cross-linking of gelatin on the dissolution properties of SGCs will also be discussed.

## 1. Introduction

Drug compounds with poor aqueous solubility present several challenges with respect to formulation and dosage form design, mainly because solubility influences the amount of drug that can be dissolved and hence available for absorption. Drugs with low aqueous solubility, especially Biopharmaceutics Classification System (BCS) class II and IV drugs, tend to undergo dissolution rate-limited absorption in the gastrointestinal tract (GIT), which in turn leads to incomplete drug absorption. The literature describes several strategies to improve the solubility and dissolution of poorly soluble drugs [1]. One of the techniques proposed to solve this problem is to formulate drugs using lipid-based drug delivery systems. This type of system is employed to maintain the drug in the encapsulated state and thus keep it in the solubilized form until it is absorbed. The distinct advantage of self-microemulsifying drug delivery systems (SMEDDS) is to improve the delivery of lipophilic drugs with poor bioavailability. These systems are capable of stabilizing the active pharmaceutical ingredient (API), improving intestinal permeability, increasing bioavailability, and protecting the API from enzymatic hydrolysis and minimizing food effects [2,3,4,5,6]. Likewise, SMEDDS are capable of promoting lymphatic transport of highly lipophilic drugs [2,3,7,8] and can easily be incorporated into soft gelatin capsules (SGCs) [9]. SGCs are an ideal tool for the delivery of lipid-based systems and hence overcome the challenges presented by poorly soluble drugs. Because of this, the SGCs approach has been utilized as an oral delivery formulation strategy for the development of poorly soluble drugs [10]. Several drugs have been formulated in the form of SGCs, and some examples of commercially available drug products are presented in Table 1. Apart from the commercial drug products listed in Table 1, SGCs are also widely used in the formulation of herbal drug products [11]. The use of these products has recently increased and they make a significant contribution in the global market of pharmaceuticals [12]. Some examples of herbal SGCs drug products include omega-3 [13], curcumin [11], and hypericum extract [12]. A soft gel capsule is a one-piece hermetically sealed soft gelatin shell containing a liquid, a suspension, or a semisolid, referred to as the fill material. SGCs can also be enteric-coated for certain applications [14]. Once SGCs are formulated, they then require discriminatory dissolution methods to assess their performance. Initially, dissolution tests were developed to evaluate the rate and extent of drug release from solid oral dosage forms like tablets and hard gelatin capsules [15], resulting in hurdles that must be overcome when these methods are extrapolated to novel dosage forms like SGCs. This review focuses on challenges in dissolution method development for SGCs. A brief discussion to understand how the physicochemical and formulation factors affect the dissolution properties of SGCs will be highlighted. Likewise, the influence of cross-linking gelatin and the effect of the addition of enzymes to the dissolution media on the dissolution properties of SGCs will also be discussed.

## 2. Advantages and Disadvantages of SGCs

**Figure 1:**
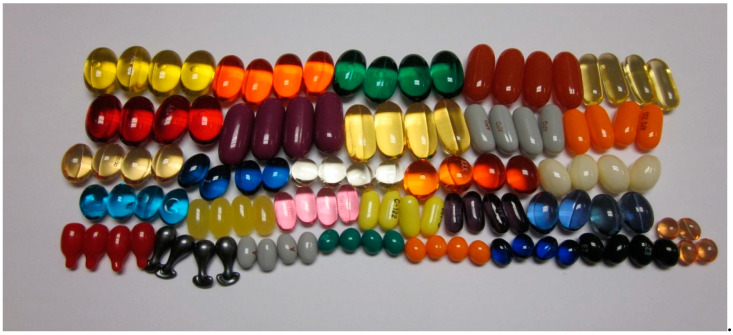
A selection of SGCs with various sizes and shapes.

SGCs offer several advantages when compared to traditional oral solid dosage forms, and their popularity as a dosage form is increasing for several reasons, that include:

(1) Consumer Preference: SGCs dosage forms were developed to conceal the unpleasant taste and odor of drugs. Compared to tablets, SGCs are more comfortable to swallow when used with water because the soft gelatin capsule is self-lubricating [16,17]. SGCs look more appealing and enjoyable to consumers as they can easily be produced in various shapes, sizes, and colors (Figure 1), and different drug delivery system like chewable softgels [18] and meltable softgels [19].
(2) Technical Advantages: SGCs have high dosage accuracy and uniformity [16,17] as well as higher consistent manufacturing requirements and product stability. It is possible to deliver an API with a higher degree of accuracy and greater consistency between different manufacturing lots due to more accurate compounding, blending, and dispensing of liquid fill materials. SGC products tend to have higher stability as the entire encapsulation process can be done under inert conditions to protect drugs against oxidation and degradation. This is especially important for drugs that are subject to hydrolytic and oxidative degradation.
(3) Safety Aspects: The tight sealing of the gelatin shell protects the fill material from air and environmental contaminations. The shell can be formulated to block ultraviolet (UV) as well as visible light. Also, SGC formulation helps to avoid dust handling contaminations and enhances operator safety [17].
(4) Bioavailability Advantages: SGCs can increase the bioavailability of poorly soluble drugs by improving solubility and enhancing absorption within the GIT [20,21]. Water-insoluble drugs are formulated in form of SGCs using lipophilic vehicles as a portion of fill material, greatly enhancing uptake of such drugs within the GIT [22].

Despite these advantages, SGCs are not a first-line oral dosage form of choice for most pharmaceutical companies due to the following reasons:

(1) SGC technology is believed to be relatively expensive to produce, and this can increase prices to consumers. Many pharmaceutical companies do not have the specialized equipment necessary to fill SGCs, and most of them rely on contract laboratories/manufacturers for their supplies.
(2) Unlike solid dosage forms, SGCs can also be affected by humidity and microbial contamination. This can cause product stability issues if the medications are not kept in sealed containers or a cool and dry place.
(3) Depending on the nature of the drug that is dissolved within the lipophilic vehicle, there is a chance that the drug could migrate into the shell of the capsule. This migration can cause issues during absorption within the body, as the release rate of the drug would be altered. Likewise, SGCs are not generally capable of holding water-based liquids, as there is a possibility that the medication could diffuse out of the soft-gelatin capsule [16,17].
(4) Another issue concerning the use of soft gelatin drug products is the fact that some groups have dietary restrictions that prevent them from consuming animal products found in SGCs. Gelatin is primarily made from bones, skins, and other parts of animals such as pigs and cows. Because capsule shells are made from animal parts, many vegetarians also opt not to use them. Due to this, there is emerging technologies on animal-free substitute gelatin capsules made from seaweed extract or other sources, but they are generally more expensive and harder to find [23].
(5) Gelatin is extremely water-soluble, which helps it dissolve in the body. The downside of this property is that SGCs are sensitive to heat and humidity. In hot or humid climates, capsules may stick together or even break open before consumers have a chance to use them [17,24].
(6) Alkaline or acidic solutions are not good candidates for soft gelatin fill because they can cause hydrolysis and leakage of the gelatin shell unless their pH is adjusted to neutral [20].

## 3. Influence of Physicochemical Properties of Gelatin on the Dissolution of SGC Drug Products

**Figure 2:**
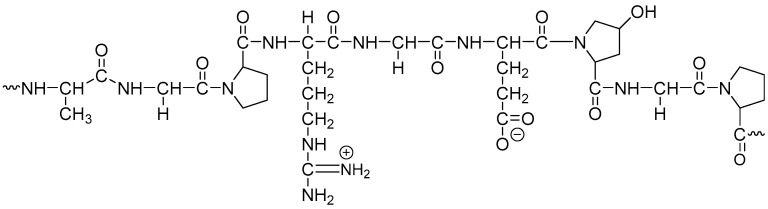
Representative chemical structure of gelatin. Reproduced with permission from [28]. Wiley-VCH GmbH, 2007.

Gelatin (Figure 2) is a natural product obtained by the partial hydrolysis of collagen derived from the skin, white connective tissues, and bones of animals [25], and is a generally recognized as safe (GRAS) food ingredient. The most abundant source of gelatin is pig-skin [26]. Gelatin is classified based on their method of preparation. Gelatin derived from an acid pre-treatment is known as Type A, while gelatin derived from an alkali pre-treated process is known as Type B. There are no plant sources of gelatin; however, gelatin substitutes are available [23,27].

In pharmaceutical applications, it is important to understand how the physicochemical properties of gelatin can affect the dissolution rate of SGCs. Gelatin that is used for pharmaceutical applications must fulfill the requirements set by the official pharmacopoeias such as the United States Pharmacopeia (USP) and the European Pharmacopeia (Ph. Eur.). Gelatin must have the ability to set at a fast rate into ribbons of defined thickness and mechanical characteristics sufficient to tolerate all the processes on the encapsulation machine. The strength and functionality of gelatin are derived from its triple helix structure in aqueous solution, with the critical functional property being the ability to form a thermo-reversible gel. This triple helix structure is the foundation of the intrinsic strength of the seal and shell of the capsule.

Considering these aspects, the technologically relevant gelatin parameters are gel strength/Bloom, viscosity, solubility, melting point, setting point, particle size, and molecular weight distribution. The main physicochemical factors that are relevant to the dissolution of SGCs are briefly discussed below.

### 3.1. Gel or Bloom Strength

The essential quality of a gelatin solution is known as gel or Bloom strength, which is a function of gelatin molecular weight, concentration of gelatin in the solution, pH, and viscosity [29,30,31,32]. It increases with a rise in gelatin concentration, average molecular weight, and as the pH of the gel approaches neutrality. The Bloom strength is defined as the weight in grams that, when applied with a 12.7 mm-diameter plunger, will produce a depression exactly 4 mm deep in a matured jelly containing 6.7% *w*/*w* of gelatin in water [33]. Bloom strength is usually between 50 and 300; however, the gelatin used in the manufacture of SGCs have Bloom strengths of approximately 150–200 [17]. Bloom strength greatly influences the clarity and color of the gelatin capsule. For a given SGCs, there is an inverse relationship between the Bloom strength and the dissolution rate, with the higher Bloom strength resulting in a lower dissolution rate and vice versa [34,35]. This relationship holds if other parameters are kept constant.

### 3.2. Gel Viscosity

Viscosity is likely the second most crucial property of gelatin, and depends on its grade, Bloom strength, and molecular weight [36,37,38]. Its value generally increases with increasing Bloom strength. For use in SGCs, the gelatin solution should ideally have a viscosity of 2.8 to 4.5 mPas at 60 °C, depending on the gelatin type [17]. Gelatin solutions used for encapsulation of active pharmaceutical ingredients (APIs) must have enough viscosity stability, also known as retention of viscosity, so that they can stay at the ribbon casting temperature for the duration of staging. The thickness of soft gelatin capsule is normally a function of the viscosity of the gelatin solution. Hence, based on the explanation given in point number 1 above, there is a significant relationship between gelatin viscosity, Bloom strength, and dissolution rate of SGCs.

### 3.3. Molecular Weight Distribution

Gelatin has a molecular weight distribution between 10 and 400 kDa [39,40,41,42]. High proportions of low molecular weight gelatin lead to shortened holding time and poor capsule seals, while high proportions of high molecular weight gelatin cause higher viscosity and encapsulation problems. The data available have shown that the higher the cross-linking of gelatin, the higher the molecular weight, and this is inversely proportional to the dissolution rates of gelatin [43]. A molecular weight profile provides useful information on the functional performance of the gelatin, such as encapsulation and dissolution [39,40,41,42].

### 3.4. Setting Point

The setting point of gelatin is the temperature at which it forms a gel, and this temperature is dependent on the thermal and mechanical history of the gelatin [44]. Higher setting temperatures are achieved when the solution is cooled down slowly. In practice, the gelatin used for soft gelatin capsule applications must set at a temperature significantly lower than its melting point. In addition, the gelatin ribbon must have sufficient Bloom strength to soften fast enough under the heat of the wedge and become elastic to expand in the die pocket by fill material. This process is known as gelation. The gelatin setting properties will ultimately influence the rate of dissolution [45].

Other crucial characteristics of gelatin that can affect dissolution include melting point, solubility, particle size, and chemical composition. Gelatin is not soluble in alcohols and non-polar solvents. In cold water, gelatin swells, and when heated above the melting point, the hydrated gelatin will rupture and go into solution [46]. Well-defined and controlled gelatin particle size is crucial for reproducible dissolution and de-aeration times of the molten mass.

The choice of the gelatin type and grade for soft gelatin encapsulation will depend on technological requirements or capabilities, consumer preference, and pricing. For pharmaceutical products, medium Bloom limed bone gelatins or blends of limed bone and pig-skin are commonly used, with a certain preference for limed bone gelatin in the USA [17]. Low-viscosity pigskin or acid bone gelatins with a Bloom strength of about 200 are often used for the encapsulation of hygroscopic formulations and water-sensitive drugs. Standard gelatin formulations contain less water and dry faster to improve product stability. Furthermore, mixtures of gelatin with Bloom strengths of 100 and 150 have been suggested to be suitable for the formulation of chewable SGCs [47].

In general, the ideal gelatin for SGCs has the following preferred characteristics: Bloom strength of 150 to 200, well-controlled and defined viscosity of about 2.8 to 4.5 mPa, particle size, gelatin type, and molecular weight distribution [17].

## 4. Manufacture of SGCs

**Figure 3:**
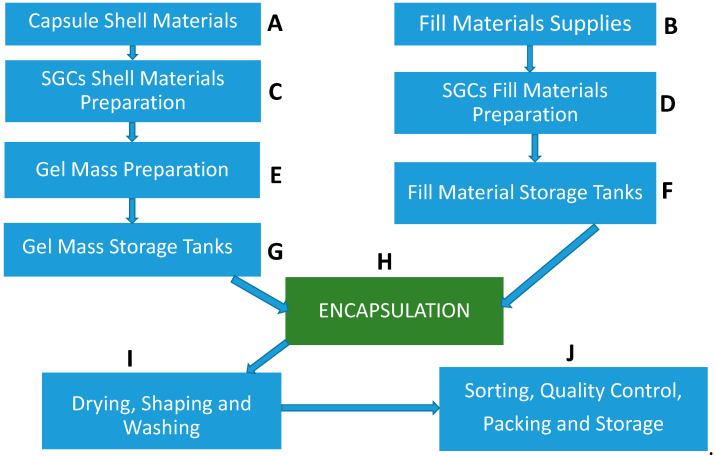
Schematic representation of (**A**–**J**) different steps in the manufacturing of SGCs.

Before discussing shell and fill formulation of SGCs, an overview of the industrial SGC filling process is briefly highlighted in this section. These steps are critical as they can influence the dissolution characteristics of SGCs. For details of the manufacturing process of SGCs, readers are referred to Gullapalli [48]. The main steps during the SGC manufacturing process are as follows:

(1) Raw materials such as raw gelatin, plasticizers, and purified water are mixed under vacuum conditions at a temperature of about 70 °C depending on gel formulation (Figure 3C,D). Any undissolved gel is removed by filtration.
(2) All compounds required for a specific formulation are added and mixed under appropriate vacuum and nitrogen blanket conditions. During the mixing process, samples are taken from a different area of the vessel to ensure homogeneity of the mixture and for viscosity control. The vessel size depends on the development of the commercial stage of a drug product; for example, gelatin melting tanks can range from small scale (e.g., 100 L) to large scale (e.g., 1200 L), while gelatin service tanks can range from 50 L (lab scale) to 300 L (large scale) [49]. These vessels are connected to a mill to reduce particle size to below 180 µm for the encapsulation process.
(3) The mixture is stored in the receiver containers (Figure 3F,G).
(4) When fill material and gel are ready, the encapsulation with soft gelatin will start (Figure 3H). Gel and fill material are connected to the encapsulation machine through heated tubing and under the nitrogen atmosphere. After encapsulation, SGCs enter a tumbler for the initial drying process. Then, they are stored in a drying tunnel for several days, depending on formulation and capsule size, to dry further. Most of the moisture is removed during this stage, and capsules’ moisture content and hardness is measured at the end of this step.
(5) After capsules are dry to the desired level, they are sorted and graded (Figure 3I,J)—grading is especially critical and important for blister production.
(6) Capsules are polished with a cloth soaked with isopropanol for a defined period and then with a dry cloth for the same amount of time. In the end, capsules are printed and packaged (Figure 3J).

## 5. Shell and Fill Formulation of SGCs

When developing a SGC formulation, the possible interaction between the fill material and the gelatin shell must be considered. These interactions may happen during manufacturing, drying, or over the shelf-life of the product. Interactions could include a chemical reaction between fill components/and their oxidization or degradation products, and the shell or physical interaction such as migration of fill material into or through the shell and vice versa [50,51]. The extent of these interactions depends on many factors. These include the amount of each component in the shell and fill formulation. One of the most well-known problems of the shell formulation is associated with APIs or excipients containing reactive functional groups such as carbonyl, which can result in gelatin cross-linking [43,52,53]. Cross-linking will be discussed in detail later.

Important parameters of the fill material to consider during the filling operation may include particle size (in the case of suspension formulation), temperature, and viscosity of fill material. Fill material must be viscous enough so that displacement pumps do not show stringing and have precise dosage. The temperature range is usually between 37 and 40 °C for proper sealing. In addition to these operational conditions, a vehicle for fill formulation development should meet the following benchmarks:

(1) It should be able to dissolve API completely and prevent precipitation of excipients and API during manufacturing and throughout the shelf-life period.
(2) It must be able to stabilize fill material and be compatible with gelatin shell formulation.
(3) It is desired that the developed fill formulation optimizes the physical and chemical stability of the API.

**Figure 4:**
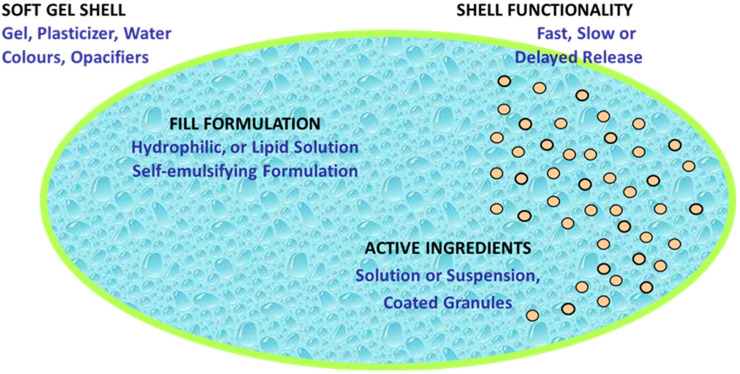
Schematic representation of the soft gel functional dosage form.

Interestingly, the fill composition of SGCs has evolved from lipophilic to hydrophilic solutions/suspensions and complex self-emulsifying systems due to demand for new chemical entities and biopharmaceutical formulations. Traditionally, SGCs comprise of lipophilic oil fill materials such as soya bean oil or castor oil, and lipophilic APIs such as vitamins A, D, E, and K, as well as herbals which do not react with shell composition at all [48,54,55]. Figure 4 shows a wide range of materials that can be filled into SGCs.

Hydrophilic SGC fill formulations are based on polyethylene glycols (PEGs), and this includes low molecular weight PEGs and closely related compounds such as methoxy polyethylene glycols, propylene carbonate, propylene glycol, glycerin, ethyl alcohol, and diethylene glycol monoethyl ether [48,56,57]. Use of PEGs, especially with a low molecular weight such as 200 and 300, and water should not exceed more than 10 percent of fill composition as they act as plasticizers as well. PEG-based formulations are used to improve solubility, disperse low-dose and high-potency drugs, and improve bioavailability.

Self-emulsifying lipophilic systems are new formulation approaches that contain a non-ionic surfactant(s), such as Tween or lecithin, and probably co-solvent(s) [48,58,59]. These systems are usually developed to increase the bioavailability of poorly soluble drugs. This type of formulation enhances the dissolution and absorption of a drug because upon release in GI fluids, fill material spontaneously produces small droplets with an average size less than 100 nm [60,61,62].

**Figure 5:**
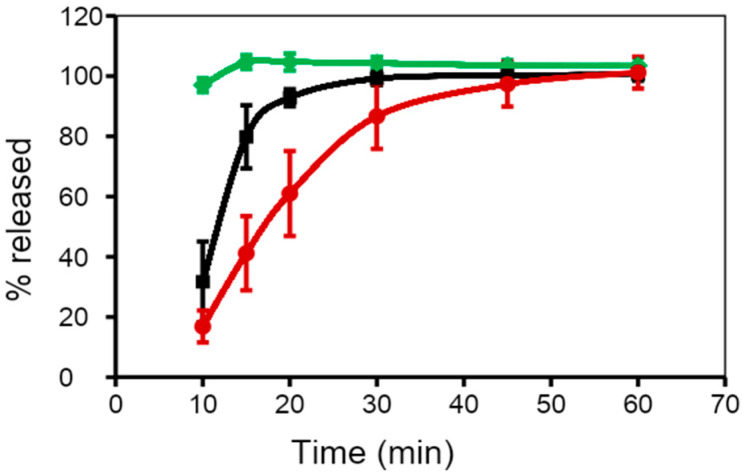
Dissolution profiles of loratadine drug products formulated using different concentrations (in %) of the fill materials using the USP dissolution apparatus 1 (basket) at 75 rpm in 0.1 N hydrochloric acid (HCl) in purified water, (■) MCM to MCT (50:50), (■) MCM to MCT (75:25), (●) MCM to MCT (100:0). Each data point represents the average ± standard deviation (*n* = 6). Reproduced with permission from [63], Dissolution Technol., 2006.

Another approach in the fill formulation is the addition of suspending, thickening, or viscosifying agents, to inhibit precipitation of dispersed materials and preserve the homogeneity of fill formulation throughout the encapsulation process [59]. Suspending agents have hydrophobic properties and high viscosity, which helps to improve the stability of SGCs by minimizing migration of water from the shell into the fill and the diffusion of water-soluble compounds from the fill into the shell. In general, the suspending agents used for PEG-based fill formulations include higher molecular weight PEGs such as PEG 6000, cellulose polymers, and mixtures of mono-, di-, and tri-glycerides/mono- and di-fatty acid esters of polyethylene glycols. It is imperative to point out that the physical and chemical properties of the drug and the fill materials in SGCs play a significant role in controlling the rate of dissolution of SGCs. Figure 5 shows the dissolution profiles of SGCs of a Biopharmaceutics Classification System (BCS) II drug formulated using different ratios of medium-chain triglycerides (MCT) and mono- and di-glycerides mixtures (MCM). The data presented in this figure confirm the effect of fill characteristics on the dissolution of SGCs formulation of poorly soluble drugs.

Shell formulation for SGCs typically consists of gelatin, plasticizer(s), water, and other minor additives such as colorants, flavors, opacifiers, sweeteners, and possibly sugar, preservatives, and in rare cases, even active ingredients [48]. Water serves as a solvent to make a liquefied gelatin formulation with a pourable viscosity at 60–70 °C. To obtain protection for light-sensitive ingredients or more consumer-appealing appearance, the shell can be formulated with pigments such as titanium dioxide and iron oxides [64].

To achieve elasticity of soft gelatin capsule shells, the use of a plasticizer is required [65,66]. The plasticizer provides mechanical stability of the shell during and after drying by offsetting induced stresses due to shrinking of the capsule shell. A plasticizer should be able to reduce the glass transition temperature of the gelatin blend. Like other components of the shell, the type and concentration of plasticizer depends on the fill material formulation, use and storage conditions, and size and shape of the capsule. To avoid product problems and issues such as dissolution and cross-linking, possible interactions of plasticizer with fill material should be anticipated [67]. The percent of plasticizers ranges from 15% to 30% *w*/*w* of the total wet mass of a shell formulation during encapsulation [48]. A wide range of plasticizers for use in the formulation of SGC shells is available, and the concentration and type of plasticizers used have an impact on the characteristics of the final drug product. Glycerol, sorbitol, and sorbitol/sorbitan solutions are the most frequently used plasticizers for SGCs [17,68]. Glycerol is a highly efficient plasticizer with low volatility that forms a stable thermally reversible gelatin [17]. It is used mostly for soft capsules to be stored at ambient conditions. Glycerol/sorbitol combinations are suitable for more rigid capsules used in warm and humid environments and capsules containing oxygen-sensitive fill material. In contrast to glycerol, which is a direct plasticizer, sorbitol is an indirect plasticizer and acts as a moisturizing agent. In addition, propylene glycol and low molecular weight polyethylene glycol, such as PEG 200, are frequently used as plasticizers. Propylene glycol has superior plasticizing capability in comparison to glycerol and sorbitol; however, it also dissolves gelatin, making its application limited [17,68]. The rate of SGCs rupture or disintegration will depend on the composition of the gelatin shell and aging conditions.

Depending on fill and shell composition, there might be challenges in the formulation of SGCs. For example, fill compositions with hydrophilic components are sometimes more challenging because they may diffuse to the shell during the manufacturing and drying processes as well as during product shelf-life [17,48,51,66,69,70]. To solve migration issues and stability of products, the composition of shell and fill materials should be designed in such a way as to minimize migration. Several solutions have been proposed and used to overcome this issue, including (1) use of higher-Bloom strength to reduce the initial water content in the capsule shell, (2) using glycerol/sorbitol or sorbitol/sorbitan blends instead of glycerol alone as the plasticizer, and (3) coating drug particles to prevent discoloration of formulations due to browning reaction between the active ingredients [17]. As an example, migration of material has been reported in microemulsion fill formulations comprising hydrophilic co-solvents such as propylene glycol and ethanol, and surfactants [50,71]. Propylene glycol and ethanol can facilitate material migration from fill to shell or vice versa by their softening and volatilizing properties, respectively [72,73]. Issues caused by propylene glycol can be solved by using it as a plasticizer component in the shell. The advantage of this technique is the reduced amount of water needed in the formulation due to the lower viscosity of propylene glycol compared to glycerol. Volatilization of ethanol can be solved by (1) using solvent-tight packaging materials such as an aluminum blister and (2) replacement of glycerol by higher polyols such as xylitol or sorbitol. In some cases, the only way to overcome this issue is to replace ethanol with another co-solvent that does not show any diffusion into the shell, such as dimethyl isosorbide [72,73].

## 6. Dissolution Methods Development and Considerations for SGCs

Noyes and Whitney first documented the study of the dissolution process in 1897 as a field of physical chemistry, which later was mimicked in pharmacy due to its importance in drug administration [74]. The dissolution of solid dosage forms attracted attention as the realization of the importance of drug dissolution concerning bioavailability was identified in the 1950s with the understanding that only dissolved drugs can diffuse through the human body [74,75,76,77,78]. Poor drug solubility and low dissolution rates potentially lead to insufficient availability of the drug at the site of action and subsequent failure of the in vivo therapeutic performance. This is independent of the fact that the drug could be an ideal structure for the target site. Essentially, if the drug is too insoluble, it can never reach its target site, and it will be of no therapeutic relevance. Characterization of the dissolution of a drug from a given dosage form is critical for the successful development of a drug product. This section discusses the current state-of-the-art of SGCs dissolution and various practical concepts of developing dissolution methods for SGCs.

### 6.1. Dissolution Definition

**Figure 6:**
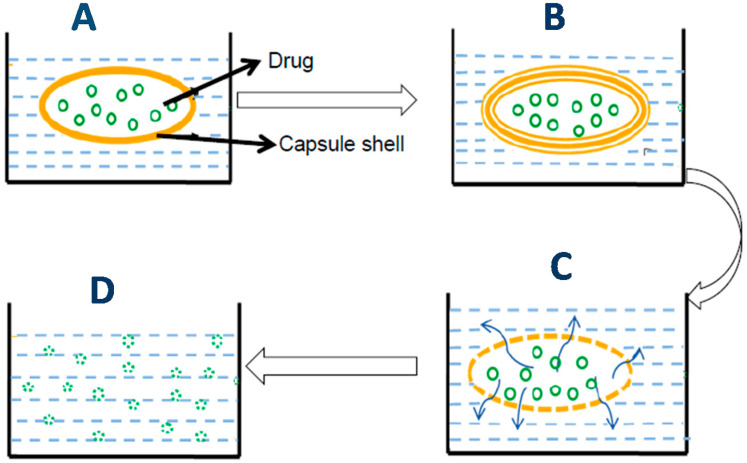
A schematic diagram showing different stages of drug dissolution from SGCs. (**A**) The initial state of the SGC, (**B**) swelling of the SGC shell, (**C**) rupture of the SGC shell to release the fill materials, (**D**) dispersion and dissolution of the drug in the dissolution medium. Reproduced from with permission from [63], Dissolution Technol., 2006.

Dissolution testing is an official test used for evaluating the rate of drug release from a dosage form into the dissolution medium or solvent under standardized conditions of liquid/solid interface, temperature, paddle speed, or solvent composition. Dissolution testing has become important in measuring the in vitro rate and extent of API release from different dosage forms, including SGCs. Dissolution can be described as a process by which molecules of a solute (e.g., API) are dissolved in a solvent to form a solution. The in vivo effectiveness of a dosage form depends on its ability to release the drug for systemic absorption. SGCs dissolution goes through three main steps, the first one being swelling and rupture of the gelatin shell, followed by release and dispersion of the fill material, and finally, the dissolution of the active ingredient(s) in the dissolution medium (Figure 6). These processes occur in series, and thus the slowest step determines dissolution rate of the SGCs. The slowest step in this case controls the overall rate and extent of drug absorption. However, this varies from drug to drug. For poorly soluble drugs, especially BCS II and IV, their dissolution will be a rate-limiting step in the absorption process. On the other hand, for drugs that have high solubility, their dissolution will be rapid, and rate and extent of absorption can be affected by other factors, e.g., membrane permeability, enzymes degradation in the GIT, or first pass metabolism.

### 6.2. Dissolution Rate

The dissolution rate of a drug product in each solvent is defined as the rate of transfer of individual drug molecules from the solid particles into the solution as individual molecules, and it can be expressed as the concentration of dissolved API for a given time interval. The rate of dissolution can vary depending on the form of API, e.g., the amorphous form usually has rapid dissolution compared to crystalline forms of API [79,80].

Another concept that needs to be introduced here is the drug release phenomenon. Drug dissolution rates and drug release rates are quite different. Drug release refers to the process by which the drug in a drug product is released in the dissolution medium or at the site of absorption by diffusion or dissolution of a drug product. Depending on the physical form of the API in the drug product, the release of API may be slow or immediate. As described in the previous section, dissolution is a process by which molecules of a solute are dissolved in solvent vehicles as a function of time. On the other hand, the term “release” most often refers to a much more complex phenomenon. Release encompasses capsule dissolution as one of its several steps. Upon contact with the aqueous medium, water penetrates the soft gelatin shell and at least partially dissolves the API [81]. Then, the dissolved API diffuses out through the capsule shell due to concentration gradients. Furthermore, the gelatin shell might undergo significant swelling as soon as the critical water content is reached, which will result in the rupture of the shell, followed by dispersion and eventual dissolution in the release medium. Hence, several steps are involved in the process of releasing the API from SGCs drug products, with only one of them being drug dissolution.

A critical requirement for drug products is that they release the APIs in vivo at a predictable rate [9,82,83]. The kinetics of drug release follows the release mechanism of the system, such as diffusion through the inert matrix, diffusion across the gel, osmotic release, ion-exchange, or pH-sensitive delivery systems. Among the various mechanisms involved in API release, diffusion is the principal release mechanism, and it takes place at varying degrees in every system. Solute release models in physical chemistry preceded the development of drug delivery systems by many years [77,78]. In 1961, Higuchi introduced a mathematical model of drug release for diffusion-controlled systems [84]. The author analyzed the release kinetics of an ointment, assuming that it is homogeneously dispersed and is released in the planar matrix and the medium. According to the model, the release mechanism is proportional to the square root of time [85]. This model is recommended for the initial 60% of the release curve due to its approximate nature. In late 1969, Wang published an article considering the two independent mechanisms of transport, Fick’s law, and polymer relaxation on the molecules’ movement in the matrix [86]. Then, Peppas, in 1985, introduced a semi-empirical equation, power law, to describe drug release from polymeric devices in a generalized way [87,88].

### 6.3. Solubility

Another important thermodynamic property in a discussion of dissolution processes is solubility, which may be expressed in several ways, including but not limited to molarity, molality, mole fraction, mole ratio, and parts per million. As an illustration, for the case of a drug molecule, consider an excess amount of solid that is exposed to the solvent phase at a defined temperature and pressure. In the equilibrium state, the number of drug molecules going into the solution equals the number of drug molecules which re-precipitate. Under these conditions, the solution is saturated with drug molecules and the concentration of dissolved drug under these conditions is defined as the “equilibrium drug solubility” (specific to the given temperature and pressure) [89]. It is important to assure that the solid phase present at the beginning of the experiment remains unaltered after reaching thermodynamic equilibrium during any solubility experiment. It is worth mentioning that, when particle size or the presence of additives, or the pH modifies the intrinsic solubility, this is usually reported as “apparent solubility” to distinguish it from the equilibrium value. In order to avoid the inconsistency in solubility data reporting, the size of filters used in the separation of dissolved drug particles must be stated.

### 6.4. Disintegration and Rupture Tests

The disintegration test is considered as one of the performance tests for the immediate release dosage forms [90]. As per the USP <701>, disintegration is defined as “the state in which any residue of the unit, except fragments of insoluble coating or capsule shell, remaining on the screen of the test apparatus or adhering to the lower surface of the disk, if used, is a soft mass having no palpably firm core” [91]. The requirements of disintegration are met if all test units have completely disintegrated or if not fewer than 16 of a total of 18 units tested are disintegrated within a predetermined time period. This does not imply complete solution of the API or the drug product.

However, the USP General Chapter <2040>, Disintegration and dissolution of dietary supplements, accepts a rupture test as a performance test of SGCs if the capsule content is semi-solid or liquid [92]. The rupture test is performed using apparatus 2, as described under General Chapter Dissolution <711>, at a rotation speed of 50 rpm in 500 mL of immersion medium for a duration of 15 min. As per USP <2040>, the requirements are met if all of the SGCs tested rupture in not more than 15 min”. If 1 or 2 of the SGCs rupture in more than 15 min but not more than 30 min, the test is repeated on 12 additional SGCs: not more than 2 of the total of 18 capsules tested rupture in more than 15 but not more than 30 min. For SGCs that do not conform to the above rupture test acceptance criteria, the test is repeated with the addition of papain to the medium in the amount that results in an activity of not more than 550,000 units/L of medium or with the addition of bromelain in the amount that results in an activity of not more than 30 gelatin-digesting units/L of medium [92]. Almukainzi et al. [93] compared the rupture and disintegration tests of SGCs of amantadine, ginseng, flaxseed oil, pseudoephedrine hydrochloride, and soybean oil. Their data showed that neither rupture test nor disintegration test was advantageous over the other. However, rupture test reached the endpoint quicker compared to the disintegration test. In another study, Bachour et al. [94] evaluated the suitability of the rupture test for stability studies of SGCs containing oil-based oral multivitamins. Their study showed that the rupture test was sensitive to stability conditions, and that the commercial drug products passed the rupture test. However, all long-term stability samples failed the rupture test using tier 2 conditions. This indicates that the rupture test may be suitable for assessing the performance of some drug products, but this will depend on the properties of fill components.

### 6.5. Practical Concepts of Developing a Dissolution Method

Dissolution testing is used throughout drug product development as an indicator of drug product performance. During formulation development, dissolution testing is used to demonstrate the release and uniformity of a dosage form in a simulated environment. Once the performance is established for the product, this information is used periodically during stability to determine if the characteristics of the product are changing in such a way that the product continues to or stops performing as required. Often, the performance of a drug product in dissolution shows physical behavior; however, it does not necessarily indicate performance in vivo. Therefore, correlation between dissolution and pharmacokinetic data can be used to demonstrate if dissolution testing has the ability to predict drug performance. This is referred to as establishing in vitro–in vivo correlation (IVIVC) [95].

The purpose of this section is to give an overview of the practical concepts of developing dissolution test methods for SGCs. It is important to understand that the dissolution of a product requires a number of physical changes to take place. Unlike other typical solid dose forms, SGCs must first reach the point where the integrity of the gelatin is compromised and the outer shell ruptures to allow release of the fill material. Following this, the fill components must disperse within the media to allow the active ingredients to either enter solution or distribute evenly throughout the media (Figure 6). The challenge is that the capsule shell is very sensitive to its environment and can change relative to hardness, cross-linking, and seam integrity, which can all play a role in perceived dissolution changes when in fact they are changes in rupture time. Therefore, it is essential to develop a dissolution strategy that accounts for differences in the integrity of the capsule shell as well as changes in the fill material.

Dissolution methods development are labor-intensive processes even with careful technique and practice. It is important to invest time in developing a procedure that can be efficiently executed on a routine basis and repeated robustly. Dissolution tests are required by the Pharmacopeias to determine the release of the drug from the dosage form in an environment with a pH from 1.2 to 7.4. For example, USP <711> [96] requires a two-step dissolution method for enteric-coated solid oral dosage forms that demonstrates coating integrity in an acidic environment, usually 0.1 N HCl, followed by exposure to a neutral pH environment, preferably with a phosphate buffer, where the first step of dissolution method provides information about the coating quality and the potential for coating failure. The United States Pharmacopeia (USP) and the U.S. Food and Drug Administration (FDA) provide guidelines on the development and validation of dissolution procedures [96,97]. Most of these guidelines are for solid oral dosage forms like tablets and hard gelatin capsules; however, one cannot extrapolate these methods to SGCs without proper assessment. The choice of dissolution method should be based on the dosage form and the fill characteristics of SGCs. Table 2 shows the common USP dissolution apparatus used in dissolution testing.

Developing a discriminating dissolution test for SGCs requires special considerations and knowledge of gelatin and fill material properties and factors influencing them. Several factors affect the dissolution behavior of SGCs and subsequently affect the development of dissolution procedures. These factors include physical properties of the gelatin shell, physical and chemical properties of the fill material, chemical interaction between the gelatin shell and fill components, and moisture exchange between the shell and the fill material. In particular, moisture exchange can potentially result in brittleness of the gelatin shell, and chemical interactions between the shell and fill could result in gelatin cross-linking.

Two key considerations in the design and development of dissolution methods are the solubility of the active ingredient and solution stability of the SGCs. To establish a suitable medium, several dissolution media should be evaluated to identify the one that achieves appropriate sink conditions. Sink conditions can be defined as the volume of medium that is at least three times the saturated solubility of the API, with the lowest quantity of designated surfactant. These studies allow optimization and observing the amount of surfactant that is needed to solvate the fill material within a time that is relevant to the dissolution test. It is more reasonable that a dissolution result reflects the properties of the API under the sink conditions; however, a medium that fails to provide sink conditions may be acceptable by the USP if it is appropriately justified. Likewise, when choosing the medium, the effect of additives such as acid and salt concentration, buffer counter-ions and co-solvents, and types of enzymes and their activity must also be evaluated and justified, if used. The solubility improvement of the API depends on various factors, including the nature of the surfactant and the fill material, temperature, pH, and ionic strength. This relationship should be understood for different surfactants and compounds before executing the dissolution experiment.

Typical media for dissolution studies include: dilute hydrochloric acid (0.1 N), buffers in the physiologic pH range of 1 to 7.5 (i.e., phosphate, acetate, or citrate), simulated gastric or intestinal fluid (with or without enzymes), water, and surfactants such as Tween, Brij 35, Triton, polysorbate 80, cetyl trimethyl ammonium bromide (CTAB), sodium lauryl sulfate (SLS), and bile salts [100]. Some SGC formulations may contain a matrix or API that is not soluble in water or acidic environment and consequently, does not meet sink conditions in aqueous solution. In these instances, surfactants with a justified concentration may be added to the dissolution medium. The choice of surfactant and its concentration in relation to solubility and physical stability of the API is critical and must be optimized, understood, and justified. The addition of surfactant should reflect changes in the formulation and interactions among fill components and may shed light on the in vivo behavior of the SGCs.

Surfactants play a role in dissolution by replacing water molecules on the particle surface, which reduces interfacial tension between the solution and the surface [101]. Amidon et al. has proposed that the use of media containing surfactants is a suitable method for solubilizing such drugs because various surfactants are present in the GI fluid, e.g., bile salts, lecithin, cholesterol and its esters [102]. They consist of two distinct components, hydrophilic and hydrophobic, and are categorized into four groups according to the charge on the hydrophilic group: anionic (e.g., sodium lauryl sulfate (SLS)), cationic (e.g., cetyl trimethyl ammonium bromide (CTAB), zwitterionic (e.g., alkyl betaine) [101], and non-ionic (e.g., Tween and Triton) [103,104]. Dissolution media containing cationic surfactants are better able to discriminate dissolution rates of acidic fill materials, while anionic surfactants differentiate better for basic fill materials. SLS has been reported to be the most commonly used surfactant in dissolution studies [100]. Solubility and dissolution rate enhancement by the surfactants are a function of surfactant concentration and the size of a micelle, and its stability, all of which can be related to the critical micelle concentration (CMC) [105]. The CMC is defined as the minimum concentration of a surfactant’s monomer at which it aggregates to micelles and is characteristic for each surfactant. A lower CMC value for a given surfactant means the micelles are more stable [106]. Furthermore, the knowledge of the molecular structure of the surfactant can provide information on the size of the micelles.

**Figure 7:**
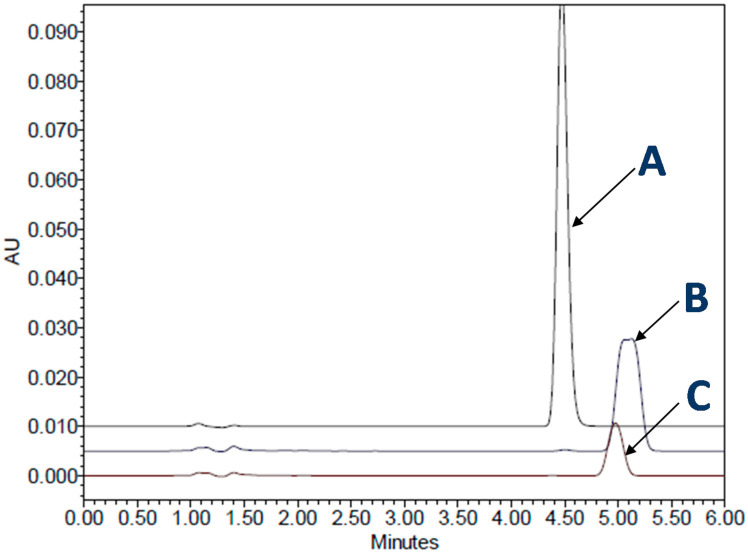
HPLC chromatograms of loratadine in 0.1 N HCl in purified water + 0.1 % SLS: (**A**) represents loratadine standard in 0.1N HCl, (**B**) loratadine soft gelatin capsule in 0.1 N HCl + 0.1% SLS dissolution media after 10 min, and (**C**) loratadine soft gelatin capsule in dissolution media with SLS after 60 min. Chromatographic conditions were done as described by Damian et al. [63].

It is important to note that the addition of surfactant to dissolution media can sometimes cause a decrease in the dissolution rates of some drug products, and in some instances can also distort drug peaks during high-performance liquid chromatography (HPLC) analysis (Figure 7). In a previous study [63], it was found that an immediate-release SGC, containing a poorly soluble drug, loratadine, showed peaks distortion in the presence of SLS. A similar observation of a decrease in the dissolution of gelatin capsules with SLS at lower pH has also been reported by other research groups [107,108].

The development of simulated fluids for dissolution testing requires understanding of the physiological conditions of the GIT. It is important to note that the GIT is complex and has a regional dependence drug absorption [109]. Several physiological factors that can affect the dissolution process in vivo include: surfactants in gastric juice and bile, viscosity of the GI contents, GI mobility patterns, GI secretions, pH, buffer capacity, and co-administration of fluids or food [110]. Vertzoni et al. [111] developed a fasted-state simulated gastric fluid (FaSSGF) containing sodium taurocholate, lecithin, and pepsin at pH of 6.5 in order to assess its importance for the in vivo dissolution of lipophilic compounds. The authors concluded that simulation of the gastric content was essential in order to assess the absorption profile of lipophilic weak bases. An overview of the composition of the common in vitro bio-relevant dissolution media is provided by Klein [112] and Galia et al. [113]. Likewise, simulated dissolution media must take into account the developmental changes in gastrointestinal fluid composition because these can result in variations in luminal drug solubility between children and adults. Therefore, evaluating age-specific changes in GI fluid parameters (i.e., pepsin concentration, bile acids, luminal viscosity, pH, osmolality, etc.) is very important in order to define the composition of bio-relevant dissolution media in pediatrics [114]. Furthermore, aged population with medical conditions such as hypochlorhydria and achlorhydria have elevated gastric pH [115]. Therefore, simulated dissolution media in this population may need to be adjusted to reflect this increased pH.

**Figure 8:**
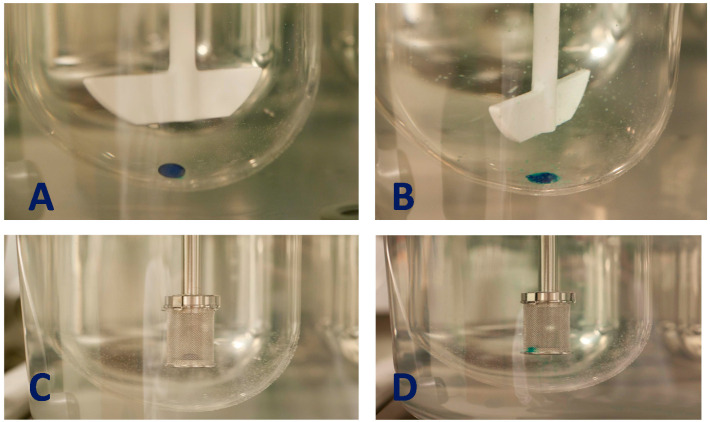
Dissolution of loratadine SGCs using apparatus 1 and 2. (**A**,**B**) represent time 0 and 5 min, respectively (apparatus 2). Oil droplets from the soft gel capsules can be seen in the dissolution media in (**B**). The visual observation of dissolution of the same drug product using USP apparatus 1 is presented by panels (**C**,**D**) at time 0 and 5 min, respectively. No pellicle formation or clogging of the basket mesh could be observed.

The selection of dissolution apparatus is another critical step in the dissolution evaluation of SGCs, as the mixing efficiency of fill material contents with media is very much influenced by the agitation hydrodynamics, particularly to variables such as paddle rotation speed. The two commonly used methods for evaluating the dissolution properties of SGCs are the paddle and basket methods (Figure 8).

A basket apparatus has the advantage of enclosing SGCs. This method may be selected if SGCs are filled with a material that has a specific gravity less than that of water, where baskets prevent the SGC and its components from floating in the medium. One common problem observed using the basket is that during the dissolution experiment, the soft gel shell may disintegrate into a soft and sticky mass that can clog the basket’s mesh, generating high variability in the results. Additionally, if the fill material is hydrophobic, i.e., an oil-based fill, dispersion into fine droplets that can pass through the basket’s mesh may not take place, giving rise to a delay in dissolution that is not representative of the true properties of the SGCs. To mitigate this problem, an alternative would be using a basket with larger pores, i.e., 20 or 10 mesh sizes [116]. Pillay and Fassihi used a rotating basket method to evaluate the dissolution of lipid-based SGCs of nifedipine. Their data showed that, after six hours of dissolution test, most of the viscous oily fill formulation was still entangled within the baskets and this led to the dissolution failure [55]. This was attributed to using the standard dissolution basket with pores size of 40 mesh, combined with inappropriate hydrodynamic conditions within the basket. However, when the dissolution test was repeated using a re-designed dissolution apparatus, in this case, nifedipine SGCs showed the best dissolution profiles.

The paddle method constitutes about 70% of the dissolution methods used by FDA-approved commercial drug products [100]. This method does not use a mesh basket to contain the capsules, and so a common initial problem observed in this method is the floating of the SGCs to the surface of the dissolution medium once it breaks. In these instances, wire coils, also known as sinkers, can be used to enclose the soft gels and hold them on the bottom of the vessel [117]. This allows the fill to be better exposed to the medium (upon shell rupture) and helps to prevent the capsule from sticking to the vessel walls. The shape and size of the sinker should be selected carefully as it can impact the dissolution process, especially in cases where SGCs swell when they encounter the dissolution medium. In previous study, it was shown that the dissolution rate obtained using the paddle method was faster, highly variable at lower time points than those obtained using the basket. In contrast, the data collected using the basket dissolution apparatus showed that the method was more selective and had less variation in terms of API release profile [63]. Table 3 shows examples of SGCs that are commercially available and their dissolution methods. Other research groups have evaluated the feasibility of using the USP III in evaluating the dissolution of SGCs. Monterroza and Ponce De León [118] developed a discriminating dissolution method of SGCs containing an oily suspension of micronized progesterone. They compared the dissolution profiles generated using USP 1, 2, and 3. After preliminary tests, USP 1 and USP 2 methods did not reach the target of releasing more than 85% of the API in less than 90 min. However, USP 3 showed promising prospect of releasing more than 85% of the API in less than 90 min in the presence of 250 mL of 4% of SLS in pH 6.8 phosphate.

In some cases, such as coated SGCs, a two-step or two-tier dissolution technique must be developed [120,121,122]. The purpose of this method is to assess the integrity of the coating in the acidic conditions of the stomach and measure the drug release in lower parts of the GIT, which have near-neutral pH conditions. Manually performing the two-step dissolution test is labor-intensive and requires well-trained analysts. For example, it requires pre-heating the second medium solution, adjusting the medium by adding the second part of the solution as well as adjusting and confirming pH for six vessels within 5 min. Typically, there are two approaches towards medium modification known as medium-addition or medium-exchange. For example, both approaches may start with an acidic step, such as 0.1 N hydrochloric acid, for a certain period, followed with a buffer step, such as phosphate buffer at pH 6.8. The specific time is chosen as needed for the individual drug product. While using either approach, the pH adjustment must be accomplished in a controlled and reproducible manner via pre-heated media. The operation of adding and adjusting the pH must be done within 5 min [123]. Zhao and co-workers described a two-step dissolution method using medium addition and paddle apparatus, in which the surfactant Tween 80 was included in the media to enhance the solubility of the API in the first stage [124]. The developed dissolution method was able to discriminate against the changes in composition, manufacturing process, and stability of the drug product. When developing a two-step dissolution procedure, several factors must be carefully examined to establish a suitable medium. The most critical step is to carefully evaluate different media to identify the one that achieves the sink conditions. The fill material may have a pH-dependent solubility, so an evaluation of the solubility of the compound in both the acidic and neutral media must be made. For instance, 0.1 N HCl and 50 mM pH 6.8 phosphate buffers are commonly used media.

The medium-addition technique, which is used for a two-step dissolution for enteric-coated capsules or two-tier dissolution testing, uses paddle or basket apparatus. This approach requires the addition of a relatively small amount of medium to each vessel in a short time. Generally, the common dissolution volumes used are in the range of 500 to 1000 mL, with 900 mL being the most commonly used in the FDA-approved drug products [100]. However, the dissolution volumes should be defined by the sink conditions. To develop a robust two-step dissolution method which can be transferred to quality control, a medium addition method is preferred where a volume of, e.g., 200 mL, can be added to 700 mL initial volume to adjust pH, and then add the surfactant, or enzyme, depending on the soft gelatin capsule drug product [124]. Furthermore, an accurate volume of the medium must be added to ensure that a volumetric error does not occur. Likewise, media addition must consider the final desired pH of the final volume. This technique is less invasive for the SGCs and is easier to conduct in a short time when running multiple batches. This approach is also less labor-intensive and allows for higher sampling throughput during the experiment run. For use in enteric-coated drug products, the API should be soluble up to the specification level in the medium of the first step to be able to detect a failure in the coating. For example, if the specification level for the first step is not more than 10% released, then this medium must be able to dissolve at least 10% of the active ingredient in the soft-gelatin capsule drug product. If the fill material is not soluble in the first-step medium, a surfactant may be added to solubilize at least 10% of the API in the fill material [124]. For use in two-tier dissolution, the fill material would require the surfactant to be present to meet solubility requirements, but also needs the enzyme to overcome the cross-linking.

For the medium-exchange approach used for enteric-coated capsules, the acid medium is drained after the first step, and a full amount of pH 6.8 buffer that has been equilibrated at similar conditions is added to the same vessel for the buffer stage. The dosage form should be undisturbed during the medium change. The complete medium replacement method resembles the medium-addition approach in that the capsules are first introduced to an acidic medium. At the end of the first step, a sample for analysis is taken, and then the dosage form is removed from the acidic conditions. Removing technique of dosage form depends on the type of dissolution apparatus. The dosage forms may be manually moved from one vessel to another. Alternatively, the entire vessel containing the acid could be removed and replaced with another vessel containing the buffer, and the dosage form is transferred to the new vessel. The quality of the SGCs dosage form is ensured by meeting the USP acceptance criteria for the acid stage, i.e., less than 10% of the API is released from the drug product during the first step of the developed dissolution technique, and therefore, the coating is considered to have passed the acid-step test. If each unit release is not less than Q + 5% for the buffer stage, then the soft gel dosage form has passed the second step of dissolution [125]. Q represents the amount of an active ingredient dissolved in the dissolution medium, expressed as a percentage of the labelled content. To overcome the challenges of manual manipulations of adding the buffer solutions and adjusting the pH during the two-step dissolution testing, other research groups have developed semi-automated dissolution systems for these measurements [125]. The media exchange technique is challenging for SGCs, especially if the capsules have softened due to the liquid exposure, soaking alone will cause some softening but may not cause the rupture of the capsule. Therefore, the transfer of the capsule or media removal without disturbing the shell may be difficult due to mechanical stress.

The European Medicines Agency (EMA) has developed its own guidance on in vitro dissolution tests for immediate-release drug products [126]. In dissolution guidance, EMA describes specifications for the quantity of active substance dissolved in a specified time, which is expressed as a percentage of API on the product label. The goal of the guidance is to set specifications to ensure batch-to-batch consistency and highlight possible problems with in vivo bioavailability. The guidance for solid immediate-release (IR) drug products from the European Pharmacopoeia (Ph. Eur. 5.17.1) has some differences compared with the FDA specifications. From a pharmaceutical perspective, the European Pharmacopoeia (Ph. Eur.) states that IR formulations should normally achieve in vitro dissolution of at least 80% of the drug substance within not more than 45 min. However, based on the USP guidance, in general, 85% or more of the drug substance should be released within 30 to 45 min.

Dissolution methods for SGCs must also consider the aspect of age-related gelatin cross-linking influencing the dissolution performance. The USP <711> permits the use of a two-tier assessment of hard and *SGCs* when evidence of cross-linking is present. Evidence of cross-linking usually occurs based on visual observations during the performance of the dissolution testing. This is based on the fact that the USP general chapters on dissolution <711> as well as disintegration and dissolution of dietary supplements <2040>, allow the addition of various enzymes based on pH of the dissolution medium when hard or SGCs and gelatin-coated tablets do not conform to the dissolution or to resolve potential cross-linking issues specifications [127]. Cross-linking evidence can come in the form of poorly dissolving gelatin shell or pellicle formation, which appears as a sac surrounding and containing the fill material after the shell is dissolved (see Section 8). To overcome cross-linking, the two-tier dissolution test would involve the addition of proteolytic enzymes such as pepsin, papain, bromelain, or pancreatin to the dissolution media and repeating the dissolution [128]. These enzymes effectively digest the peptide bonds between the amino acids making up the gelatin strands in the shell. The use of enzymes for dissolution must be done with care, as the enzymes require significant mechanical mixing to get into solution, are minimally stable in solution, and can be impacted by other components of the media, such as surfactants. If a protein denaturing surfactant [129] is used in the media, a two-step tier 2 method must be performed. The first step involves the dissolution of the capsule shell using media containing an enzyme and no surfactant as a pre-treatment step. After the capsule shell is dissolved, media containing surfactant is added to complete the dissolution and solubilization of the fill and active pharmaceutical ingredient. It was observed that using the digestive enzyme while conducting the dissolution study and afterward using the surfactant showed a better effect in the two-tier method [130].

Another important aspect that is worth discussing regarding dissolution of SGCs is the concept of an in vitro–in vivo correlation (IVIVC). This is normally used to establish a relationship between an in vivo response (e.g., amount of drug absorbed) and an in vitro physicochemical property of a dosage form. The main objective of this concept is to make sure that the in vitro properties of two or more batches of the same drug product are performing similarly under in vivo conditions. Hence, this relationship is essentially important in guiding drug development and drug approval processes that are designed to mimic the in vivo drug release. There have been various studies on IVIVC of SGCs and some have shown good correlations. Meyer et al. [53] assessed whether the changes in the in vitro dissolution of hard and soft gelatin acetaminophen capsules, as a result of gelatin cross-linking, are predictive of changes in the bioavailability of the capsules under in vivo conditions. Their data showed that the in vitro rate of dissolution of hard and SGCs decreased due to cross-linking. On the other hand, the bioequivalence studies showed that both hard and SGCs, which failed to meet the USP dissolution specification in water, but complied when tested in SGF containing pepsin, were bioequivalent to the unstressed control capsules. Based on the plasma concentration parameters, the capsules that were cross-linked to the greatest extent were not bioequivalent with the unstressed control capsules. In another study, Nishimura et al. [131] attempted to predict the human plasma drug concentrations of SGCs containing a poorly soluble drug, arundic acid. SGCs were stored at short- and long-term conditions, i.e., 15 °C for 3 months and 25 °C (60% relative humidity (RH)) for 30 months, respectively. The authors showed that the in vitro dissolution data obtained with the dissolution medium containing surfactant (i.e., 2% SLS, pH 6.8) were more effective in predicting the drug plasma concentrations following oral administrations of the SGCs under both storage conditions. Likewise, Rossi et al. [132] developed and validated a dissolution test for ritonavir SGCs based on human in vivo pharmacokinetic data. The authors used a USP II method with 900 mL of dissolution medium containing water with 0.3%, 0.5%, 0.7%, or 1% *(w*/*v*) of SLS at rotation speed of 25 rpm. Their data showed strong level A correlation between the percent of the drug dissolved versus percent absorbed. Significant in vitro–in vivo correlation was achieved using dissolution medium containing water with 0.7% SLS. In another similar study, Donato et al. [133] reported similar results on the development and validation of a dissolution test for lopinavir, a poorly water-soluble drug, in soft gel capsules, based on in vivo data. In this work, a new formulation of lopinavir was developed and its dissolution tests validated using in vivo data. All formulations were evaluated for in vitro dissolution containing 2.3% SLS at pH 6.0 and USP 1 at 25 rpm. At these conditions, the authors showed strong level A correlations for the fraction dissolved versus fraction absorbed.

Understanding the IVIVC relationship of new formulations of SGCs will help the design of optimized dissolution methods that can be used to predict in vivo performance.

## 7. Flow-Through Dissolution Methods

Another method that has gained much interest in the characterization of in vitro release properties of SGCs is the use of flow-through cell dissolution apparatus (i.e., USP 4). This method was initially developed for modified-release oral dosage forms [75,134]. However, it is suitable for other dosage forms [135,136,137]. The USP 4 method has several advantages that include [96,99]: easy to switch between different dissolution media with different pH, provision of sink conditions, easy in vitro–in vivo correlation characterizations, and applicable to a wide range of drug formulations, e.g., pellets, microspheres, tablets, microcapsules, hard and SGCs, implants, creams, ointments, suspensions, drug-eluting stents, and suppositories [99]. Likewise, sampling is easy because there is a continuous extraction of the drug sample from the release medium. The fresh dissolution media continuously passes through the samples in the flow cell, and this is important, especially for maintaining the sink conditions of poorly soluble drugs.

**Figure 9:**
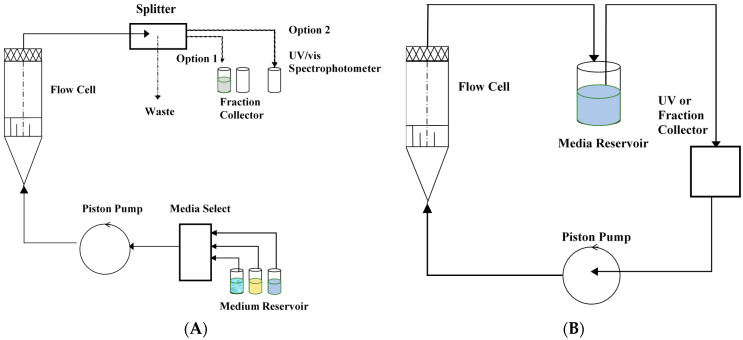
Sketch of a USP 4 flow-through system showing open loop (**A**) and closed loop (**B**) configurations.

In the flow-through cell (Figure 9), the flow of the dissolution medium is controlled by a pump from a temperature-controlled reservoir. The reservoir provides a fresh dissolution medium, which then passes through a flow-through cell that is equipped with a filter on top to prevent the passage of any undissolved particles. To provide a laminar flow of the media through the cell, the bottom part of the cell is conical and filled with glass beads. The drug product to be tested is placed on a holder or within the glass beads, and the dissolution medium is allowed to flow from the bottom. For optimal results, the recommended flow rate through the cell is between 4 and 16 mL/min [96]. There are some variations in the design of the USP 4 apparatus, and there is a possibility of having an open or closed system configuration. The drug content in the dissolution media is analyzed by HPLC in the collected fractions or analyzed automatically during the continuous flow of the dissolution media through the UV spectrophotometer. Drug content can also be evaluated using continuous UV fiber optic, where more frequent data points can be generated with reduced costs [138]. Using UV fiber optics improves accuracy in measurements and eliminates sampling errors.

Drug release from SGCs using the USP 4 method can be affected by several factors. Apart from the factors listed in Section 4, drug release can also be affected by liquid flow rates, area of the flow-through cell, initial drug quantity, and drug concentration [139]. Some reports have suggested that the USP 4 is not suitable for SGCs formulations, especially those with lipid products because the oil in the fill formulations can sit at the top of the cell and clog the system [75]. However, the flow-cell design can be modified depending on the characteristics of the drug product to be tested. Hu et al. compared the dissolution of SGCs containing a poorly soluble amine drug using the USP 2 and the USP 4. In an attempt to overcome the problem of SGCs formulated with lipids that clog the system, the flow-through cells were designed in such a way as to work differently from the standard flow-through cell [140]. The method was adapted to handle the problems of oil fills in filters, and their data were encouraging as the USP 4 was more discriminative compared to the USP 2. In another study, Neisingh et al. [141] characterized the dissolution of SGCs containing testosterone undecanoate in oleic acid using the flow-through method. The in vitro dissolution data showed a good correlation with in vivo release profiles, confirming the usefulness of this method in “in vitro” dissolution and “in vivo” correlation studies.

## 8. Effect of Cross-Linking on the Dissolution Properties of SGCs

**Figure 10:**
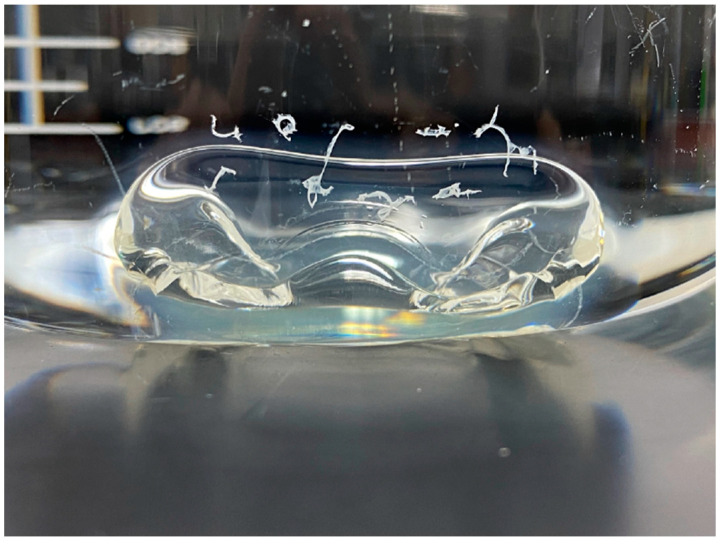
Pellicle formation due to cross-linking of SGC during a rupture test.

There are several reports of decreased dissolution rates of the SGCs attributed to the cross-linking of gelatin (Figure 10). Cross-linking of hard and SGCs is well-described in the literature [43,128,142], and it must be addressed during the development of SGCs because it can affect the release properties of the finished drug product. Several methods can be used to assess the cross-linking of these formulations, and one of them is by the observation of pellicles where the gelatin shell swells, becomes rubbery, and is insoluble in water. Figure 10 shows an example of a pellicle formation around SGCs. Moreover, cross-linking facilitates thin film formation during the dissolution test [143]. The mechanism of gelatin cross-linking owing to the aldehydes is well-known [48,143,144]. Briefly, gelatin contains several amino acids, including lysine (4.1%) and arginine (8.5%) [145]. On the other hand, aldehydes can form imine bonds between these amino acids [143]. Moreover, lysine and arginine can form inter- and intra-molecular reactions [146]. It was observed from nuclear magnetic resonance (NMR) studies that initially, methylol groups form in lysine residues and later in arginine residues. Subsequently, this results in the lysine–arginine and arginine–arginine cross-linking [146,147,148]. Apart from aldehydes, several other chemical compounds that may cause gelatin cross-linking and examples of chemical functional groups that can actively facilitate gelatin cross-linking are listed in Table 4. In addition to chemical initiation, cross-linking may be caused by changes in temperature and humidity or exposure to UV radiation [145].

In addition, the use of the FD and C Red # 3 and FD and C Red # 40 colorants were reported to modify the conformational properties of gelatin and formation of the insoluble form of gelatin [149]. Murthy et al. showed that the dissolution rates of the capsules with FD and C Red # 3 were decreased while stored in elevated humidity and light [150]. These dyes can interact with gelatin by hydrophobic interactions and hydrogen bonding [149]. FD and C are color additives that are subject to certification under USA Federal Food, Drug and Cosmetic Act (FD & C Act).

Another aspect of cross-linking may be caused by the presence of impurities in the fill material encapsulated within the gelatin shell. Polyoxyethylene (e.g., polyethylene glycols, methoxypolyethylene glycols, polyoxyethylene fatty acid esters, polyoxyethylene sorbitan fatty acid esters, and polyoxyl 40 hydrogenated castor oil) moieties present in the fill materials can undergo autoxidation in the presence of oxygen. This autoxidation leads to the production of reactive species, such as hydrogen or organic peroxides [160,161,162,163,164,165,166]. One example of this phenomenon is observed in SGCs, where the fill contains povidone as a viscosity and solubility enhancer [167,168]. Povidone contains peroxide impurities, and the amount increases with the presence of environmental oxygen [169]. In addition, an accelerated stability study showed that gelatin cross-linking occurs while the presence of the rayon coiler in the packaging material can produce furfural (2-furaldehyde) [154,170]. The drug molecules having carbonyl functional groups might induce gelatin cross-linking. Some of these drugs are rofecoxib, nimesulide, and macrolide antibiotics [171,172].

Another property that impacts gelatin cross-linking is increased temperature. At the elevated temperature, a condensation reaction occurs between the amino and carboxylic groups of two adjacent gelatin chains or with the same chain [173,174]. In addition to elevated temperature, high humidity can also induce cross-linking in gelatin. Albert et al. observed that aldehyde can induce gelatin cross-linking at moderate humidity between 60% and 70% [148]. On the other hand, Chafetz et al. reported that a considerable decrease in the dissolution rate of the drug, gemfibrozil, from soft gelatin capsule formulations stored at 37–45 °C and 80% relative humidity (RH) up to three months. Capsules having polysorbate 80 showed film formation after one month at 37 °C and 80% RH [152]. Similarly, nifedipine SGCs failed dissolution testing using the USP II method due to pellicle formation upon storage for 208 days at 25 °C (60% RH) and 40 °C (75% RH) [153]. In another study, dissolution of marketed nimesulide SGCs stored at 40 °C (75% RH) in the presence of UV and visible illumination for various periods showed an abrupt change in the dissolution behavior and no drug was released from the formulations after the eighth day of storage and afterward [175].

**Figure 11:**
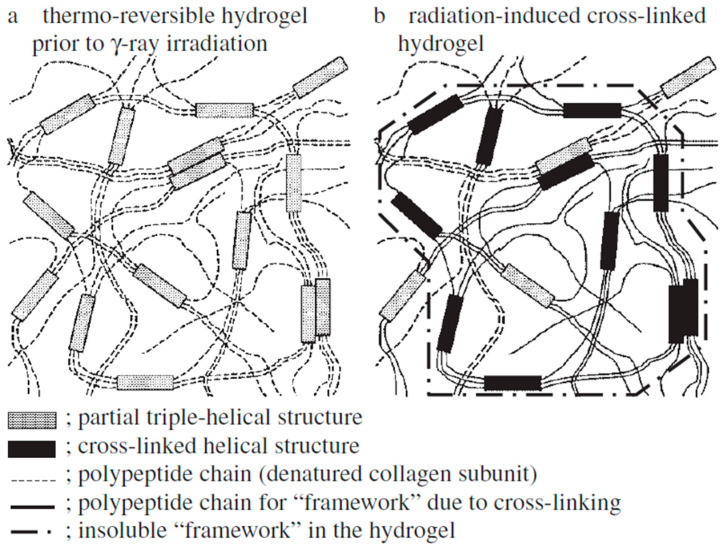
Schematic diagram of insoluble gelatin hydrogel formation by the radiation induced cross-linking. (**a**) represents thermo-reversible hydrogel prior to γ-ray irradiation, while (**b**) represents radiation-induced cross-linked hydrogel. Reproduced with permission from [176], Chemical Society of Japan, 2007.

Based on a gamma radiation study, it was observed that the application of radiation could induce gelatin cross-linking. However, irradiation of ^60^Co γ-rays on gelatin hydrogels showed that amino acids having side chains of more than two carbon atom hydrocarbon groups blocked the cross-linking of gelatin hydrogel. The authors suggested the probable model for such gelatin cross-linking as the polymerization of gelatin molecules occurring due to cross-linking of partial triple-helical structures, and consecutive connections of the polymerized gelatin molecules, including the cross-linked triple-helical structures. It was suggested that an insoluble framework of irradiated hydrogel structure is formed (Figure 11) [176].

## 9. Effect of Digestive Enzymes on Cross-linking of SGCs

**Figure 12:**
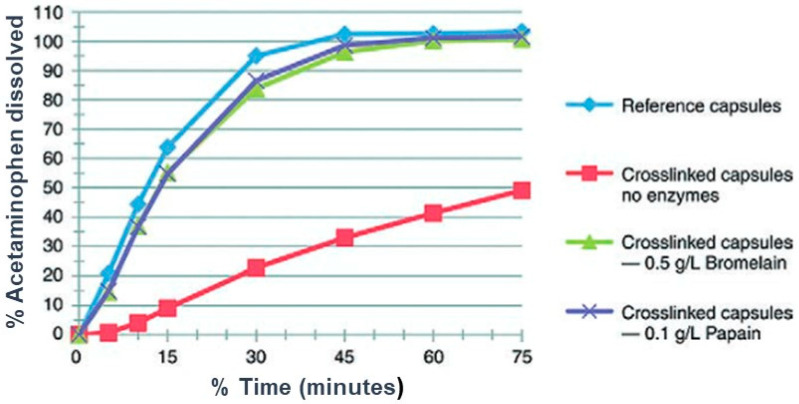
Dissolution profiles of cross-linked gelatin capsules using the enzymes bromelain and papain in pH 6.8 phosphate buffer. The solid blue line is the dissolution profile on non-cross-linked gelatin capsules (reference capsules) containing acetaminophen in pH 6.8 phosphate buffer. Reproduced with permission from [127], Dissolution Technol., 2014.

Digestive enzymes play a key role in gelatin cross-linking. Pepsin and pancreatin are present in the GIT tract and are recommended by the USP in the tier 2 method when the soft gelatin capsule fails in tier 1 without such enzymes. This is because the enzymes can potentially digest the cross-linked gelatin and promote the rupture of the cross-linked gelatin shell, and enhances the dissolution rate of the drug [96]. The recommended concentration/activity of pepsin in the dissolution media in ≤750,000 units/1000 mL for dissolution medium with a pH of less than 4 and for papain is ≤550,000 units/1000 mL of dissolution medium with a pH of between 4 and 6.8, as specified in the monograph. For the case of medium with a pH of more than 6.8, pancreatin is used with a maximum protease activity of 2000 units/1000 mL in the dissolution medium. This is well-elaborated in the General Chapter of the USP for dissolution <711> [96]. Pancreatin, consisting of many enzymes, including trypsin, amylase, lipase, and protease, is also commonly used and is capable of acting between pH 6 and 8. Interestingly, the ε-amino functional group in lysine and the guanidine group in arginine have the pKa values of 10.79 and 12.48, respectively. Therefore, these functional groups remain protonated in acidic pH and deprotonated in alkaline pH. Subsequently, at alkaline pH, they undergo reactions with aldehydes and this results in cross-linking [146]. A good example to show the role of digestive enzymes on the dissolution of cross-linked SGCs is shown in Figure 12. The authors showed the impact of bromelain and papain on the dissolution of acetaminophen cross-linked capsules. The cross-linked capsules failed the dissolution test in the absence of enzymes. However, the failure was resolved when bromelain and papain enzymes were added to the dissolution medium. The authors conducted a similar study at several pH values, and they concluded that both enzymes can be used for dissolution testing of cross-linked gelatin capsules in the pH ranges of 4 to 6.8 [127]. However, the release of the drug with both enzymes was slightly lower compared with the dissolution profile of the reference drug product.

## 10. Conclusions and Future Prospects

Recent advances have been made around formulation of poorly soluble drugs in the form of SGCs to address particular bio-performance issues, such as decreased bioavailability and increased plasma variability, by improving solubility and absorption enhancing techniques. Although SGCs provide many advantages, they also face many dissolution challenges, especially in dissolution method development. The available dissolution methods have been successfully implemented on conventional dosage forms such as tablets and hard gelatin capsules, and these methods are well-documented in official monographs. However, this is not the case with SGCs, and this challenge needs to be addressed. This is based on the fact that most individual drug candidates and drug excipients used in the formulation of SGCs possess diverse physicochemical properties requiring specific considerations. Therefore, the best approach is to develop a dissolution method that is specific to each drug product. This developed and optimized method must be capable of detecting changes in the drug product formulation, storage conditions, shelf-life, and performance. These are important points that must be considered while developing a dissolution test for SGCs:

- The solubility of the API
- Compatibility of API with soft gel fill materials
- Nature of capsule shell
- Type of surfactants in the dissolution medium
- The need for sinkers depends on whether the SGCs are floating or moving within the dissolution medium
- The design of SGCs, i.e., coated or non-coated SGCs
- Stability of the API in the dissolution medium
- Nature of fill components, i.e., hydrophilic, suspension, lipophilic, or co-solvents
- Dealing with drug products whose gelatin is already cross-linked

These points above may be changed or adjusted based on initial observations and the first set of feasibility tests. We conclude by saying that the dissolution method for SGCs needs to be drug product-specific and it may not be a good idea to generalize the method of choice. Hence, through an understanding of drug product characteristics and evaluating parameters of dissolution testing, a methodology can be established to enable batch-to-batch evaluation and evaluate in vitro–in vivo relationships.

## Figures and Tables

**Table 1:** Examples of commercially available drug products* formulated in the form of soft gelatin capsules (SGCs).

Drug	Brand and Company	Common Uses
Cyclosporine	Neoral^®^, Novartis Pharm. Corp	Prevention of organ and bone marrow transplants rejection
Dutasteride	Avodart^®^, GSK Canada	To relieve symptoms of benign prostatic hyperplasia for enlarged prostates
Calcitriol	Rocaltrol^®^, Roche Canada	Management of hypocalcemia and secondary hyperparathyroidism
Isotretinoin	Clarus^®^, Cipher Pharmaceuticals Inc.	Indicated for the treatment of severe forms of acne
Progesterone	Prometrium^®^, Merck	Hormone replacement therapy
Valproic acid	Depakene^®^, Abbott Laboratories	Antiepileptic
Testosterone	Andriol^®^, Merck Canada	Testosterone replacement therapy
Ritonavir	Norvir^®^, Abbott Laboratories	HIV ** treatment
Amprenavir	Agenerase^®^, GlaxoSmithKline	HIV treatment
Loratadine	Claritin^®^ liquid Gels, Schering-Plough Canada Inc.	Management of allergies

* Source: Information obtained from product monographs of these drug products. ** Human Immunodeficiency Virus.

**Table 2:** USP dissolution apparatus. IR—Immediate release, DR—Delayed release, ER—Extended release, ODT—Oral disintegrating tablets, ND—Non-disintegrating, CR—Controlled release [98,99].

Type of Apparatus	Principle	Common Dosage Forms
Type 1	Basket	IR, chewable tablets, DR, ER, suppositories, capsules, floating dosage forms
Type 2	Paddle	IR, ODT, chewable tablets, DR, ER, enteric-coated tablets or capsules
Type 3	Reciprocating Cylinder	CR, chewable tablets and beads
Type 4	Flow-Through Cell	ER, soft and hard gelatin capsules, powder, granules, pellets, suppositories, and implants
Type 5	Paddle Over Disk	Transdermal patches, ointments, and emulsions
Type 6	Rotating Cylinder	Transdermal patches
Type 7	Reciprocating Holder	Transdermal formulations, and ND oral-modified release formulations

**Table 3:** Examples of commercially available SGCs and their dissolution methods [119].

Drug Product Information	Dissolution Method
Cyclosporine (100 mg)	Apparatus 2 at 75 rpm in 1000 mL 0.1 N HCl containing 4 mg of N,N-dimethydodecylamine-N-oxide per mL
Dutasteride	Tier 1: Apparatus 2 at 50 rpm in 900 mL 0.1 N HCI with 2% (*w*/*v*) SLS. Tier 2: Apparatus 2 at 50 rpm in 0.1 N HCI with pepsin (as per USP) (450 mL) for the first 25 min, followed by addition of 0.1 N HCI with SLS (4% *w*/*v*) (450 mL) for the remainder of the dissolution test
Isotretinoin	Apparatus 1 with 20 mesh at 100 rpm in 900 mL 0.05 M Potassium Phosphate Buffer, dibasic, pH 7.8, containing 0.5% lauryldimethylamine N-oxide (LDAO)
Paricalcitol	Apparatus 1 at 100 rpm in 500 mL in 4 mg/mL of 0.4% lauryldimethylamine N-oxide (LDAO)
Ergocalciferol	Apparatus 2 at 100 rpm in 500 mL 0.5 N NaOH with 10% Triton-X-100
Lopinavir/Ritonavir	Apparatus 2 at 50 rpm in 900 mL, Tier 1: 0.05 M Polyoxyethylene 10 Lauryl Ether with 10 mM Sodium Phosphate monobasic (pH 6.8); Tier 2: same as above with not more than (NMT) 1750 USP units/L of Pancreatin
Amprenavir	Apparatus 2 at 75 rpm in 900 mL 0.1 N HCl
Loratadine	Apparatus 2 with sinker at 75 rpm in 900 mL. Tier 1: 0.1 N HCl with 0.1% Tween 20. Tier 2: 0.1 N HCl with 0.1% Tween 20 with addition of pepsin (as per USP)
Ibuprofen	Apparatus 1 at 150 rpm in 900 mL 50 mM Phosphate Buffer, pH 7.2

**Table 4:** Examples of cross-linking of gelatin due to chemicals.

Material	Reference
Aldehydes (furfural, acrolein, formaldehyde, glutaraldehyde, glyceryl aldehyde)	[43,151,152,153,154,155,156]
Imines	[143]
Ketones	[143]
Saccharides (glucose and aldose sugars)	[43]
Calcium carbonate	[157,158]
Hydrogen peroxide	[150,156]
Sulfonic acids and *p*-toluene sulfonic acid	[150,156]
Carbodiimides (1-ethylene 3-(3-dimethylamino propyl) carbodiimide hydrochloride, guanidine hydrochloride)	[155,156]
Benzene	[156]
Terephthaloyl chloride	[159]

## Data Availability

All data generated during this study are included in this manuscript.
